# Asthma and COPD overlap (ACO) is related to a high burden of sleep disturbance and respiratory symptoms: Results from the RHINE and Swedish GA^2^LEN surveys

**DOI:** 10.1371/journal.pone.0195055

**Published:** 2018-04-02

**Authors:** Stephanie Mindus, Andrei Malinovschi, Linda Ekerljung, Bertil Forsberg, Thórarinn Gíslason, Rain Jõgi, Karl A. Franklin, Mathias Holm, Ane Johannessen, Roelinde Middelveld, Vivi Schlünssen, Cecilie Svanes, Kjell Torén, Eva Lindberg, Christer Janson

**Affiliations:** 1 Department of Medical Sciences, Respiratory, Allergy and Sleep Research, Uppsala University, Uppsala, Sweden; 2 Department of Medical Sciences: Clinical Physiology, Uppsala University, Uppsala, Sweden; 3 Institute of Medicine at Sahlgrenska Academy at University of Gothenburg, Gothenburg, Sweden; 4 Department of Public Health and Clinical Medicine, Umeå University, Umeå, Sweden; 5 University of Iceland, Faculty of Medicine, Reykjavik, Iceland; 6 Lung Clinic, Tartu University Clinics, Tartu, Estonia; 7 Department of Surgical and Perioperative Sciences, Umeå University, Umeå, Sweden; 8 Department of Occupational and Environmental Medicine, University of Gothenburg, Gothenburg, Sweden; 9 Centre for Clinical Research, Haukeland University Hospital, Bergen, Norway; 10 Institute of Environmental Medicine, Karolinska Institutet, Stockholm, Sweden; 11 Department of Public Health, Section for Environment, Occupation and Health, Aarhus University, Aarhus, Denmark; 12 Department of Occupational Medicine, Haukeland University Hospital, Bergen, Norway, Centre for International Health, University of Bergen, Bergen, Norway; 13 Department of Occupational and Environmental Medicine, Sahlgrenska University Hospital, Gothenburg, Sweden; National and Kapodistrian University of Athens, GREECE

## Abstract

**Background:**

The term Asthma and COPD Overlap (ACO) describes a condition where asthma and COPD overlap. We aimed to investigate associations between ACO and insomnia and respiratory symptoms, and to investigate the prevalence of ACO and the characteristics of subjects with ACO in two Northern European population studies.

**Methods:**

The study comprised 25 429 subjects aged ≥ 40 years who participated in one of two Northern European general population surveys. Both surveys included questions on asthma, COPD, respiratory and sleep-related symptoms, including difficulty initiating sleep, difficulty maintaining sleep, early-morning awakening, and excessive daytime sleepiness. ACO was defined as having both self-reported asthma and COPD.

**Results:**

The prevalence of ACO was 1.0%. The group with ACO had a higher prevalence of both insomnia and respiratory symptoms than subjects with only asthma or COPD. Having ACO was independently associated with a 2–3 times higher probability of having sleep-related symptoms as compared with the group without asthma or COPD, after adjustment for age, sex, BMI, smoking history and educational level (adjusted odds ratio 2.14–3.36, 95% CI).

**Conclusion:**

Subjects with ACO have a high prevalence of insomnia and respiratory symptoms. To our knowledge, this is the first study to assess the association between sleep-related symptoms and ACO.

## Introduction

Asthma and Chronic Obstructive Pulmonary Disease (COPD) can overlap and converge, causing subjects to suffer from symptoms of both conditions. The term Asthma and COPD Overlap (ACO) has been developed to describe this condition. GOLD and GINA guidelines suggest a descriptive definition: Asthma-COPD overlap (ACO) is characterised by persistent airflow limitation with several features usually associated with asthma and several features usually associated with COPD. ACO is thus, in clinical practice, identified by the features that it shares with both asthma and COPD [1].

The prevalence of ACO has been studied in cohorts of patients with either COPD or asthma, and found to be 13% to 55% [2,3]. The prevalence of ACO is estimated as being less than 10% in COPD or asthma patients younger than 50 years and over 50% among patients aged 80 years or older. The distinction between asthma and COPD becomes increasingly difficult with patient age; features of both conditions often coexist [2,3]. Subjects with ACO are more likely to be women [2,3]. There is, however, limited data available on the prevalence of ACO in general populations.

Subjects with ACO have frequent exacerbations, poor quality of life, and consume a disproportionate amount of healthcare resources compared with patients with only asthma or COPD [2,4,5]. Little is known about the underlying causes for their poor quality of life. However, asthma and COPD have both been associated with sleep disturbances [6,7] and previous studies have shown that sleep disturbances have a large negative effect on quality of life [8]. Following this line of argument, it is likely that the low quality of life in ACO found in previous studies may partly be explained by sleep disturbances [3]. There is, however, no data on sleep quality in patients with ACO.

We aimed to investigate associations between ACO and respiratory symptoms and insomnia symptoms, including difficulty initiating sleep, difficulty maintaining sleep, early-morning awakening, and excessive daytime sleepiness, and to investigate the prevalence of ACO and the characteristics of subjects with self-reported ACO in two Northern European population studies.

## Materials and methods

### Population

The participants were recruited from two ongoing population surveys in Northern Europe: the GA^2^LEN survey and the RHINE III cohort.

The Global Allergy and Asthma Network in Europe (GA^2^LEN) is a research network in the field of asthma and allergic diseases, consisting of research centres in 22 European countries [9]. In 2008, the GA^2^LEN survey was sent to a random sample of 45 000 subjects aged 16–75 years in four Swedish cities (10 000 each in Uppsala, Stockholm and Umeå and 15 000 in Gothenburg) [6]. The questionnaire included questions on COPD, asthma, rhinitis, chronic bronchitis, eczema and environmental and workplace exposure. In addition, the Swedish questionnaire also included questions on insomnia, physical activity and cardiovascular morbidity. Up to three reminders were sent and 25 610 subjects (56.9%) responded. In the present analyses, only subjects aged 40 years or older were included (N = 13 967).

Respiratory Health In Northern Europe (RHINE) is a large Northern European prospective cohort study initiated in 1990–1992, with follow-ups in 1999–2000 (RHINE II) and in 2010–2012 (RHINE III). The cohort was initially recruited as part of the European Community Respiratory Health Survey (ECRHS) [10]. Random population samples of men and women born 1945–73 completed postal, self-administered, questionnaires in the aforementioned timeframes. The questionnaires included similar questions to those in the GA^2^LEN survey. The Swedish questionnaires also included questions on sleep-related symptoms, comorbidities and women’s health. In this study, we have used data from the RHINE III cohort, where sixty-two percent (62%) of the original sample responded. In the present analyses, only subjects aged 40 years or older were included (N = 11 462).

In both studies, informed consent was obtained from each participant. The Regional Committees for Medical and Health Research Ethics West in Norway, the National Bioethics Committee in Iceland, the Research Ethics Committee of the University of Tartu in Estonia, The Regional Ethical Review Board in Uppsala, Sweden and the Scientific Committees for Central Jutland in Denmark approved the studies.

### Definitions

The variables included in the present analyses were identical in both surveys.

Asthma was defined as a positive answer to either the question ‘Have you had an attack of asthma in the last 12 months?’ or the question ‘Are you currently taking any medicine, including inhalers, aerosols or tablets, for asthma?’ [11]. COPD was defined as a positive answer to the question ‘Has a doctor ever told you that you have chronic obstructive pulmonary disease?’. ACO was defined as having both asthma and COPD according to the definitions given above.

The questions on asthma-related symptoms included wheezing or whistling in the chest, waking up with chest tightness, and waking up due to shortness of breath at any time in the last 12 months. Response options were Yes or No.

Chronic bronchitis was defined as a positive answer to the question ‘Do you cough up phlegm from your chest in the morning almost every day during at least 3 months each year?’.

The insomnia symptoms, difficulty initiating sleep (DIS), difficulty maintaining sleep (DMS), early-morning awakening (EMA) and excessive daytime sleepiness (EDS), were defined as reporting the symptom in question at least 3 nights or days per week [11].

Smoking was assessed through the question ‘Have you ever smoked one or more cigarettes per day for more than one year?’. The question ‘Have you smoked at all during the last month?’ was then used to separate ex-smokers from current smokers.

Educational level was categorized into the following three levels: nine-year elementary school, high school and university.

Body mass index (BMI) was calculated from self-reported weight and height, as weight in kilograms divided by height in meters squared.

### Subsample with spirometry

Spirometry measurements were available from 2 099 subjects (1 376 from RHINE and 723 from the GA^2^LEN cohort). Lung function and reversibility were, in both surveys, assessed through spirometry (before and after bronchodilation with 200 μg salbutamol) using the EasyOneTM spirometer (ndd Medizintechnik AG, Zürich, Switzerland). The ratio of the post-bronchodilatory forced expiratory volume in one second (FEV_1_) and the forced vital capacity (FVC) was calculated and a ratio below 0.70 was used to indicate persistent airflow obstruction (AO) [1]. Based on these results, four groups were identified: (a) controls without AO (n = 1 491), (b) diagnosed COPD with AO (n = 6), (c) diagnosed asthma without AO (n = 247) and (d) ACO with AO (n = 29). As group b was so small, it was excluded from the analyses.

### Statistical analyses

All statistical analyses were conducted using Stata 14 (StataCorp, Texas, USA). Univariate analyses were performed with the chi-square test and the unpaired t-test. Multiple logistic regression was used to calculate adjusted odds ratios (OR) and 95% confidence intervals (95% CI) for risk analyses. Interaction analyses were performed to assess a possible modifying effect of sex on ACO. A p value of < 0.05 was considered significant.

## Results

**Fig 1:**
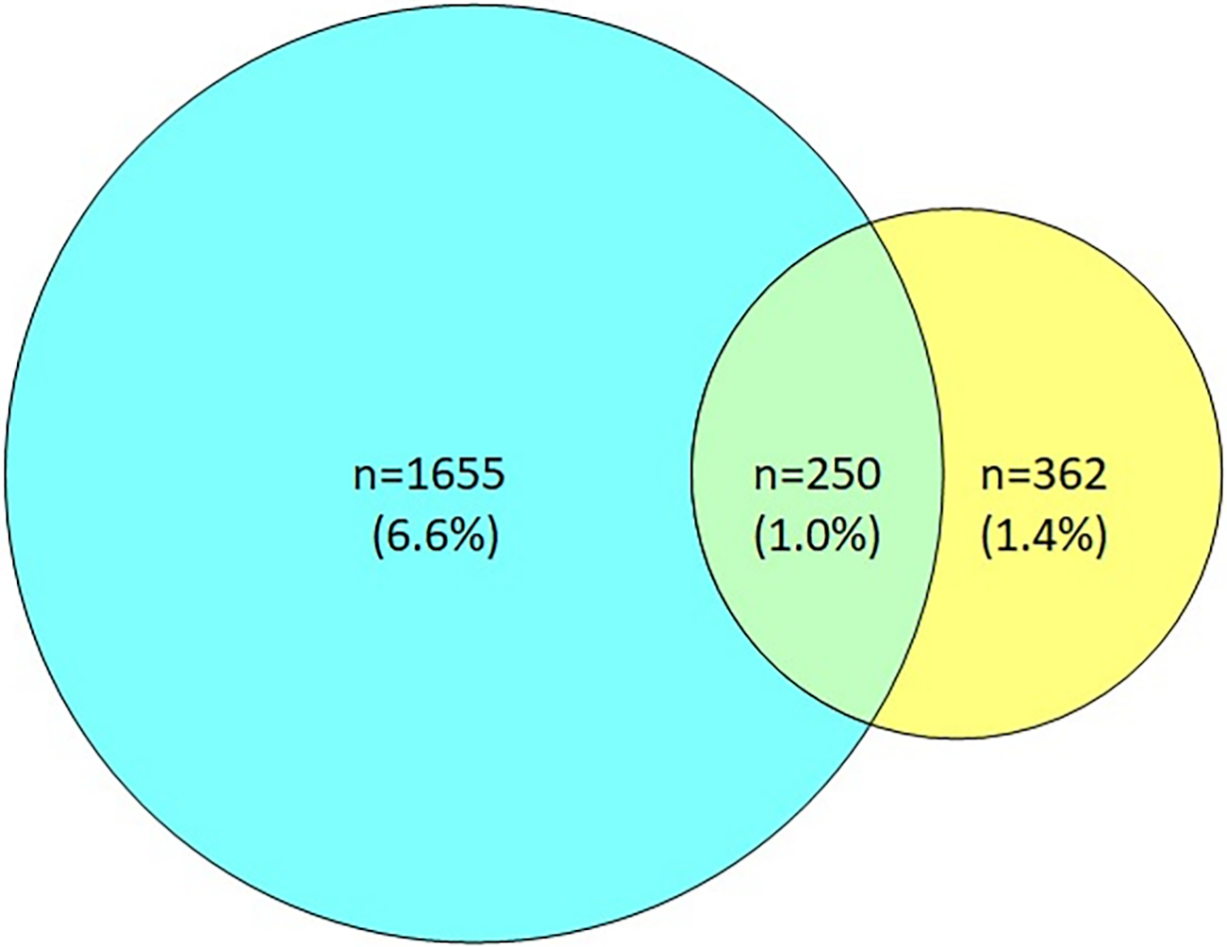
Overlap of asthma and COPD.

**Fig 2:**
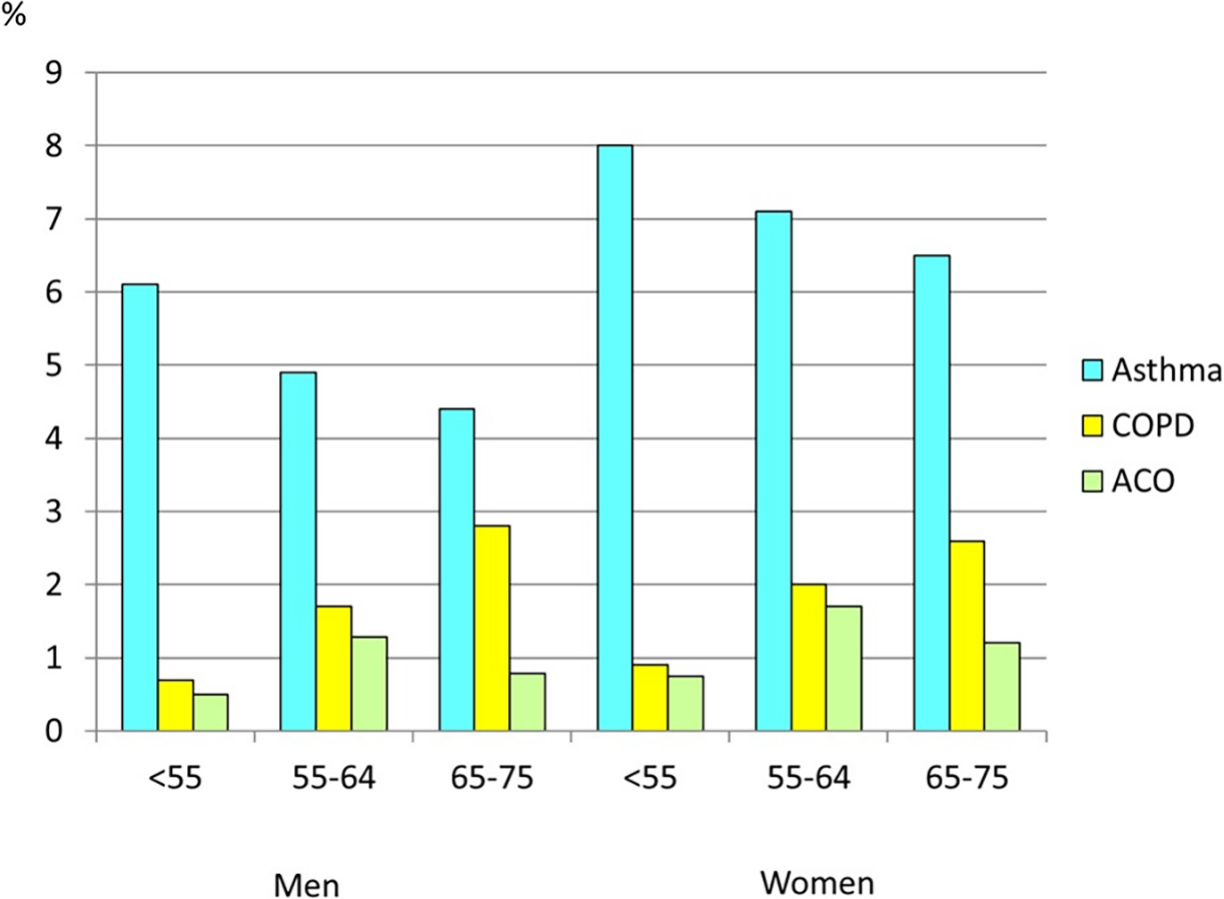
Prevalence of asthma, COPD and ACO in relation to age and sex.

In the whole cohort, 362 subjects (1.4%) reported having COPD but not asthma, 1 665 (6.6%) reported asthma without COPD and 250 subjects (1.0%) reported ACO (Fig 1). The prevalence of ACO among the participants with reported asthma was 13.1%, while the prevalence among those with reported COPD was 40.8%. The prevalence of ACO was higher in women than men in all age groups (p < 0.001) (Fig 2). The prevalence of ACO was highest in the age group 55–64 years.

The demographic characteristics of the subjects are presented in Table 1. The subjects in the groups with asthma and ACO were more often women, while the groups with COPD and ACO had the highest mean age. The group with ACO had the highest mean BMI. The group with COPD alone and the ACO group had the highest prevalence of smoking and the lowest educational level.

**Table 1:** Characteristics of the population (% and mean ± SD).

	No asthma and/or COPD n = 23 152	Asthma n = 1 665	COPD n = 362	ACO n = 250	p value
Female sex	51.7	60.2	55.8	60.0	< 0.0001
Age (years)	54.1±8.8	53.3±8.6	58.8±8.6	57.0±7.8	< 0.0001
Body mass index (kg/m^2^)	25.7±4.1	26.8±4.9	26.7±5.0	27.7±5.6	< 0.0001
Smoking history					< 0.0001
Never	48.6	49.4	17.0	18.2	
Ex-smoker	35.4	37.6	47.9	44.8	
Current	15.9	13.0	35.1	37.1	
Highest education level					< 0.0001
Elementary school	17.4	17.0	36.5	40.7	
High school	35.7	37.3	33.4	37.5	
University	46.9	45.7	30.1	21.8	

**Fig 3:**
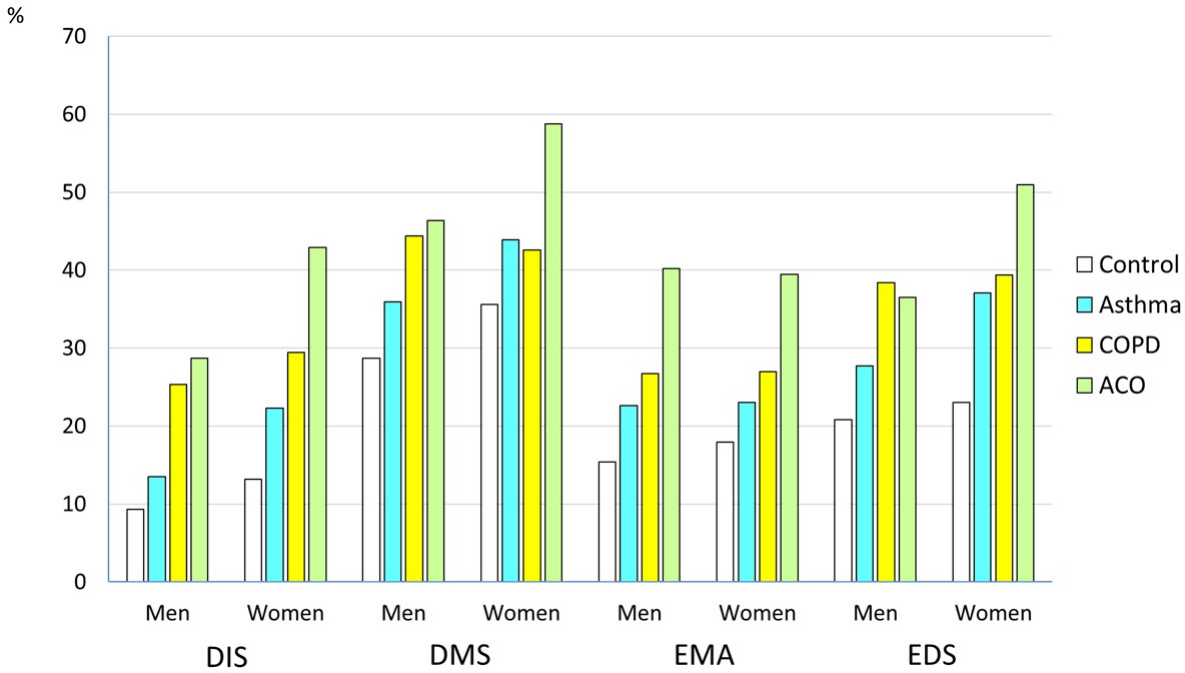
Prevalence of sleep-related symptoms in relation to asthma and COPD in men and women. (Controls: subjects with no self-reported respiratory condition, difficulty initiating sleep (DIS), difficulty maintaining sleep (DMS), early morning awakenings (EMA), excessive daytime sleepiness (EDS).

The prevalence of insomnia symptoms and the prevalence of respiratory symptoms are presented in Table 2. The group with ACO had the highest prevalence of both insomnia and respiratory symptoms. Women had higher a prevalence of insomnia symptoms (Fig 3), but the relative difference in prevalence between the ACO group and controls was similar in men and women.

**Table 2:** Sleep-related and respiratory symptoms (%).

	No asthma and/or COPD n = 23 152	Asthma n = 1 665	COPD n = 362	ACO n = 250	p value
Difficulty initiating sleep	11.3	18.8	27.7	37.3	< 0.0001
Difficulty maintaining sleep	32.3	40.7	43.4	53.9	< 0.0001
Early-morning awakening	16.7	22.8	26.9	39.7	< 0.0001
Excessive daytime sleepiness	21.9	33.4	39.0	45.2	< 0.0001
Wheeze	12.7	71.4	48.9	83.7	< 0.0001
Nocturnal chest tightness	7.8	37.7	25.0	52.4	< 0.0001
Nocturnal breathlessness	3.8	20.1	14.4	35.7	< 0.0001
Nocturnal cough	22.0	51.6	45.0	63.6	< 0.0001
Chronic bronchitis	7.8	28.6	32.7	43.3	< 0.0001

### Multivariate analyses

The strongest association with sleep-related and respiratory symptoms was seen in the group with ACO also after adjustment for age, sex, BMI, smoking history, study centre and educational level (Table 3). Interaction analyses showed no modifying effect of sex on the association between ACO and respiratory or sleep-related symptoms (p_interaction_ = 0.16–0.99). Having only asthma or COPD was also associated with a higher risk for all sleep-related and respiratory symptoms (Table 3).

**Table 3:** Sleep-related and respiratory symptoms (adjusted* odds ratio (95% CI)).

	No asthma and/or COPD n = 23 152	Asthma n = 1 665	COPD n = 362	ACO n = 250
Difficulty initiating sleep	1	1.77 (1.54–2.02)	2.15 (1.68–2.77)	3.36 (2.55–4.43)
Difficulty maintaining sleep	1	1.37 (1.24–1.53)	1.32 (1.06–1.65)	2.14 (1.65–2.78)
Early-morning awakening	1	1.45 (1.28–1.64)	1.59 (1.24–2.04)	3.00 (2.30–3.91)
Excessive daytime sleepiness	1	1.66 (1.49–1.86)	2.18 (1.74–2.73)	2.69 (1.07–3.50)
Wheeze	1	21.0 (18.6–23.7)	5.53 (4.39–6.96)	31.2 (21.8–44.7)
Nocturnal chest tightness	1	7.10 (6.33–7.96)	3.60 (2.80–4.63)	11.5 (8.82–15.0)
Nocturnal breathlessness	1	6.31 (5.47–7.29)	4.11 (2.99–5.66)	15.1 (11.3–20.1)
Nocturnal cough	1	3.58 (3.22–3.97)	2.72 (2.19–3.39)	5.70 (4.34–7.47)
Chronic bronchitis	1	5.75 (5.06–6.53)	4.41 (3.44–5.64)	9.07 (6.83–12.0)

*adjusted for age, sex, BMI, smoking history, educational level and centre.

In addition, study-specific analyses showed that the associations between ACO and sleep-related or respiratory symptoms were highly significant in both populations, although the association was stronger in the GA^2^LEN population than in the RHINE III population for most symptoms (Table 4).

**Table 4:** Association between ACO and sleep-related and respiratory symptoms in the GA^2^LEN and RHINE III populations, with participants without asthma and COPD as the reference group (adjusted odds ratio* (95% CI)).

	GA^2^LEN n = 13 967	RHINE III n = 11 462
Difficulty initiating sleep	3.74 (2.38–5.89)	3.12 (2.19–4.43)
Difficulty maintaining sleep	2.44 (1.57–3.79)	1.92 (1.39–2.68)
Early-morning awakening	3.97 (2.58–6.11)	2.46 (1.74–3.48)
Excessive daytime sleepiness	4.75 (3.06–7.38)	1.89 (1.35–2.66)
Wheeze	73.8 (33.5–162)	21.5 (14.2–32.6)
Nocturnal chest tightness	19.5 (12.3–30.9)	8.38 (6.00–11.7)
Nocturnal breathlessness	18.6 (11.9–29.0)	12.6 (8.51–18.6)
Nocturnal cough	6.19 (3.90–9.81)	5.32 (3.80–7.44)
Chronic bronchitis	10.9 (6.89–17.24)	7.97 (5.47–11.6)

*adjusted for age, sex, BMI, smoking history, educational level and centre.

#### Analyses of subsample with spirometry results

In the subsample with spirometry results, the strongest association with DIS, EMA and all respiratory symptoms was seen in the group with ACO and spirometry-defined airway obstruction (AO) after adjustment for age, sex, BMI, smoking history and educational level (Table 5).

**Table 5:** Sleep-related and respiratory symptoms (adjusted* odds ratio (95% CI)) in participants with available spirometric data.

	No asthma and/or COPD n = 1 491	Asthma n = 247	ACO n = 29
Difficulty initiating sleep	1	1.50 (0.999–2.24)	2.59 (1.07–6.24)
Difficulty maintaining sleep	1	1.18 (0.88–1.57)	0.86 (0.39–1.87)
Early-morning awakening	1	1.51 (1.07–2.11)	2.74 (1.24–6.03)
Excessive daytime sleepiness	1	1.99 (1.48–2.68)	2.08 (0.93–4.67)
Wheeze	1	16.2 (11.6–22.8)	29.8 (9.92–89.5)
Nocturnal chest tightness	1	6.40 (4.61–8.88)	18.3 (7.95–41.9)
Nocturnal breathlessness	1	5.15 (3.36–7.91)	15.0 (6.47–34.6)
Nocturnal cough	1	3.25 (2.44–4.32)	6.53 (2.88–14.8)
Chronic bronchitis	1	6.75 (4.77–9.55)	11.7 (5.15–26.5)

*adjusted for age, sex, BMI, smoking history and educational level.

## Discussion

The main finding in this community-based study is that subjects with asthma and COPD overlap have a high burden of insomnia and respiratory symptoms. These associations remained significant after adjustment for age, sex, BMI, smoking history and educational level.

The prevalence of insomnia symptoms was highest in the group with ACO overlap. This is a novel finding and has, to our knowledge, not been previously analysed, even though other studies have reported a higher burden of sleep problems in both patients with only asthma [6,12] and those with only COPD [7]. As sleep disturbances have a large negative effect on quality of life [8] it is likely that the low quality of life in ACO found in previous studies [3] is partly explained by this disturbed sleep quality. A high prevalence of fatigue in COPD has been reported in previous studies [13]. In a previous study, we found that COPD was more closely associated with sleep disturbances in men than in women [7]. In the present study, however, the association between sleep disturbances and ACO was similar in men and women, although the prevalence of sleep disturbances was higher in women in all groups.

The ACO group had a higher prevalence of wheeze than the groups with only asthma or only COPD. This is in accordance with results from the EPI-SCAN [3] and ECRHS I-II surveys [2]. The same was true for the prevalence of chronic bronchitis. This result is in accordance with data from the CHAIN cohort [14] and from ECRHS I-II [2] but not with data from EPI-SCAN [3].

The prevalence of ACO observed in our study was 1.0%. This is in agreement with previous findings from studies on general adult population samples [4]. In the present study, 13% of the asthmatics also had self-reported COPD. This is fairly similar to what has been found in other studies with participants with obstructive airway disease [15,16]. The high prevalence of ACO in those with reported COPD in the present study (41%) is probably related to that mean age was almost 60 years which is within the age interval with the highest prevalence of ACO. It is also possible that the high prevalence of ACO in the COPD group is an overestimation due to incorrect diagnosis.

As in previous studies, ACO was more common in women [3]. The groups with ACO and COPD had a higher BMI than the asthmatics and healthy controls. This was also true for the ACO groups in the CHAIN [14] and EPI-SCAN cohorts [3]. The groups with COPD and ACO had the highest prevalence of smoking. This is also in agreement with data from the EPI-SCAN [3] and CHAIN cohorts [14]. The lowest prevalence of university education was seen in the COPD and ACO groups. Data from the ECRHS I-II cohorts showed the lowest educational level for the ACO group [2]. Patients with ACO could either be patients with severe asthma that have developed fixed airflow obstruction as a result of remodelling [2] or COPD patients with asthmatic features. The fact that the characteristics of the ACO group and the COPD group were relatively similar indicate that the second of these two phenotypes dominate in the present study.

Our analysis has an inherent strength: it is the first study to describe the asthma-COPD overlap in two distinct community-based populations and to examine sleep-related symptoms in the overlap groups. Other inherent strengths are the large population samples, the consistency of our results in both population samples and the use of validated questionnaire tools.

Our study also has several limitations. Concerns could be raised about the reliability of self-reported diagnoses, both for asthma and COPD. Dissecting the phenotypes in asthma, COPD and ACO is challenging. Recall bias or physician misdiagnosis cannot be excluded. In addition, ACO is generally used as a potential diagnosis only in adults over 40 years [17] and we therefore used a lower age limit of 40 years for diagnosis in an attempt to better capture early-onset COPD. Moreover, in the GA^2^LEN and RHINE III cohorts, we only had data regarding pulmonary function in a subsample of the population. The results were, however, relatively similar when we only included participants with spirometry results. Additionally, although the response rates for the GA^2^LEN and RHINE III surveys were 57% and 62%, respectively, previous studies on the RHINE III cohort have shown that lower response rates slightly affected prevalence estimates but did not at all affect associations between predictors and outcomes [11]. Finally, the cross-sectional nature of our study is an additional disadvantage.

In conclusion, subjects with “Asthma and COPD Overlap (ACO)” suffer from a higher burden of insomnia symptoms, i.e., difficulty initiating sleep, difficulty maintaining sleep, early-morning awakening, and excessive daytime sleepiness, and respiratory symptoms, including wheeze, nocturnal chest tightness, nocturnal breathlessness and nocturnal cough. Subjects with ACO may thus need specific interventions to be adequately cared for. Women are more likely to be present in the overlap and asthma populations. Future therapeutic studies are needed to identify optimal treatment approaches for patients with concurrent asthma and COPD.
